# Reliability, costs, and radiation dose of dual-energy X-ray absorptiometry in diagnosis of radiologic sarcopenia in surgically menopausal women

**DOI:** 10.1186/s13244-024-01677-w

**Published:** 2024-04-08

**Authors:** Annechien Stuursma, Iris A. S. Stroot, Karin M. Vermeulen, Riemer H. J. A. Slart, Marcel J. W. Greuter, Marian J. E. Mourits, Geertruida H. de Bock

**Affiliations:** 1 Department of Obstetrics and Gynecology, University Medical Center Groningen, University of Groningen, Hanzeplein 1, Groningen, 9713 GZ The Netherlands; 2 Department of Epidemiology, University Medical Center Groningen, University of Groningen, Groningen, The Netherlands; 3 Department of Nuclear Medicine and Molecular Imaging, University Medical Center Groningen, University of Groningen, Groningen, The Netherlands; 4 Department of Radiology, University Medical Center Groningen, University of Groningen, Groningen, The Netherlands

**Keywords:** Costs, DXA, Observer variation, Reliability, Sarcopenia

## Abstract

**Objective:**

The aim of this study was to evaluate and compare reliability, costs, and radiation dose of dual-energy X-ray absorptiometry (DXA) to MRI and CT in measuring muscle mass for the diagnosis of sarcopenia.

**Methods:**

Thirty-four consecutive DXA scans performed in surgically menopausal women from November 2019 until March 2020 were analyzed by two observers. Observers analyzed muscle mass of the lower limbs in every scan twice. Reliability was assessed by calculating inter- and intra-observer variability. Reliability from CT and MRI as well as radiation dose from CT and DXA were collected from literature. Costs for each type of scan were calculated according to the guidelines for economic evaluation of the Dutch National Health Care Institute.

**Results:**

The 34 participants had a median age of 58 years (IQR 53–65) and a median body mass index of 24.6 (IQR 21.7–29.7). Inter-observer variability had an intraclass correlation coefficient (ICC) of 0.997 (95% CI 0.994–0.998) with a relative variability of 0.037 ± 0.022%. Regarding intra-observer variability, observer 1 had an ICC of 0.998 (95% CI 0.996–0.999) with a relative variability of 0.019 ± 0.016% and observer 2 had an ICC of 0.997 (95% CI 0.993–0.998) with a relative variability of 0.016 ± 0.011%. DXA costs were €62, CT €77, and MRI €195. The estimated radiation dose of CT was 2.5–3.0 mSv, for DXA this was 2–4 µSv.

**Conclusions:**

DXA has lower costs and a lower radiation dose, with low inter- and intra-observer variability, compared to CT and MRI for assessing lower limb muscle mass.

**Trial registration:**

Netherlands Trial Register; NL8068.

**Critical relevance statement:**

DXA is a good alternative for CT and MRI in assessing lower limb muscle mass, with lower costs and lower radiation dose, while inter-observer and intra-observer variability are low.

**Key points:**

• Screening for sarcopenia should be optimized as the population ages.

• DXA outperformed CT and MRI in the measured metrics.

• DXA validity should be further evaluated as an alternative to CT and MRI for sarcopenia evaluation.

**Graphical Abstract:**

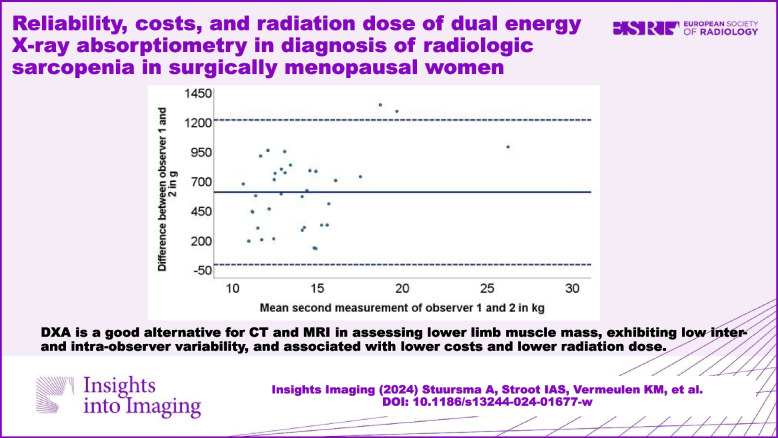

**Supplementary Information:**

The online version contains supplementary material available at 10.1186/s13244-024-01677-w.

## Introduction

Sarcopenia is a generalized muscle disorder characterized by low muscle mass, low muscle strength, and poor physical performance [1]. Age-related progressive degeneration of muscle fibers plays a central role in its pathogenesis [2], in addition to inflammation, malnutrition, lack of exercise, and imbalances in sex steroids and corticosteroids [3–7]. Prevalence of sarcopenia ranges from 3 to 24% in individuals ≥ 65 years. Sarcopenia is associated with several adverse health outcomes, such as an increased fall risk, decreased quality of life, increased disability, and increased mortality [5]. Hence, sarcopenia is becoming an increasing problem in the aging population [8, 9].

The decrease in estrogen levels in menopausal women is associated with a decline of bone mineral density (BMD), muscle mass, and strength [10]. This is especially relevant for women with an increased risk of ovarian cancer, who are advised to undergo a risk-reducing salpingo-oophorectomy (RRSO) at a young age [11] which may therefore result in earlier diagnosis of osteoporosis and sarcopenia [12, 13].

The quantitative assessment of muscle mass is crucial in the diagnosis of sarcopenia. Muscle mass can be measured with various imaging techniques to determine body composition and differentiate muscle from fat and bone tissue [1]. Examples include bioelectrical impedance analysis (BIA), magnetic resonance imaging (MRI), computed tomography (CT), and dual-energy X-ray absorptiometry (DXA) [14]. Currently, MRI and CT are considered the gold standard for diagnosing sarcopenia in a clinical setting [1]. Although MRI and CT have a high validity for assessing muscle mass, they are associated with high(er) costs and are more time-consuming [3, 15]. Additionally, CT is accompanied with a significant radiation dose [14].

An alternative for CT and MRI could be DXA whole-body composition (DXA WBC, hereafter referred to as DXA). DXA is currently only used in research settings for measuring muscle mass, since the reliability of DXA in the assessment of muscle mass in a clinical setting is unknown [16]. To the best of our knowledge, no direct comparison has been made between the reliability, costs, and the radiation dose of DXA, CT, and MRI in the assessment of muscle mass. DXA has the potential to be an alternative to CT in the clinical analysis of muscle mass and the diagnosis of sarcopenia. For that, we evaluated the inter- and intra-observer variability of DXA and compared the costs and radiation dose of DXA to CT and MRI.

## Methods

### Study design and participants

This study is considered a secondary analysis of a prospective study. It is embedded in the BENEFIT study (trial number: NL8068; Netherlands Trial Register, also available from trialsearch.who.int), which was approved by The Medical Ethical Committee of the University Medical Center Groningen (UMCG). The main aim of the BENEFIT study is to assess the long-term effects of RRSO on BMD and muscle mass. Women were *BRCA1/2* germline pathogenic variant (GPV) carriers who underwent RRSO ≥ 10 years ago before age 52 (natural menopause) and were included after written informed consent. Women were excluded if they had metastatic disease, insufficient understanding of the Dutch language, or if they were unable to visit the UMCG due to their physical condition.

### DXA

Women included in the BENEFIT study underwent DXA WBC (Hologic Horizon DXA, Marlborough, MA, USA), which was analyzed with Hologic APEX software version 5.6.0.5 (HOLOGIC, Inc, Bedford MA, United Kingdom). The system uses high and low energy X-ray beams to measure body density and body composition. The system differentiates between bone and soft tissue, the latter being differentiated into muscle and fat according to the density of the tissue [17].

### Collected data

The following clinical characteristics of the study population were collected: age, length, weight, age at RRSO, and duration of menopause. Data that were collected from the DXA scan included: total grams bone mass, total grams muscle mass, and total grams fat mass.

### Reliability, costs, and radiation dose

Reliability was assessed by calculating the inter- and intra-observer variability. For that, DXA scans were analyzed independently by two observers (I.S. and A.S.). Scans were assessed twice by both observers, each scan was analyzed four times in total. The time interval between the first and second measurement for the intra-observer variability was 2 to 3 h.

The costs of DXA, CT, and MRI were calculated according to the guidelines for economic evaluation of the Dutch National Health Care Institute (Zorginstituut Nederland) [18]. Costs were calculated separately for staff, equipment, and materials. For calculating the costs of the staff, wages were based on the ‘CAO Universitair Medische Centra 2022–2023’, the Collective Labor Agreement of University Hospitals in the Netherlands 2022–2023 [19]. For equipment (i.e., the scanners), an amortization scheme of 10 years was used. Maintenance costs for the scanners were considered 10% of its original list price at time of purchase per year. A percentage of 35% was used for housing and overhead costs. Costs for materials used were derived from invoices from the in-hospital order and delivery system.

Information on radiation dose for DXA and CT were collected from literature.

### Protocol DXA

A standardized protocol was used to make and analyze the scans, available in the Supplementary Materials. The scans were performed by trained technicians. Study participants were requested to remove all clothing, except underwear and socks. All metal items were removed before densitometry. For hygiene purposes, the table was covered with a sheath, but pillows were removed. Study participants were positioned perpendicular and in the middle of the table, with their hands next to their body, the fingers lightly spread, and the feet in slight endorotation. Participants were asked to look at the ceiling. After positioning the participant, the upper and lower borders of the scanning area were checked to ensure that the whole body was scanned. When the participant’s body was too large to fit both hands in the horizontal plane in the scanning area, the hands were placed vertically. If this did not fit, the participant was placed in such a way that the left arm was just out of the scanning field. During the analysis, the right arm was then copied to the left arm. During the scanning procedure, the DXA technicians asked patients to remain still. The scan was performed from cranial to caudal in three sequences.

**Fig. 1:**
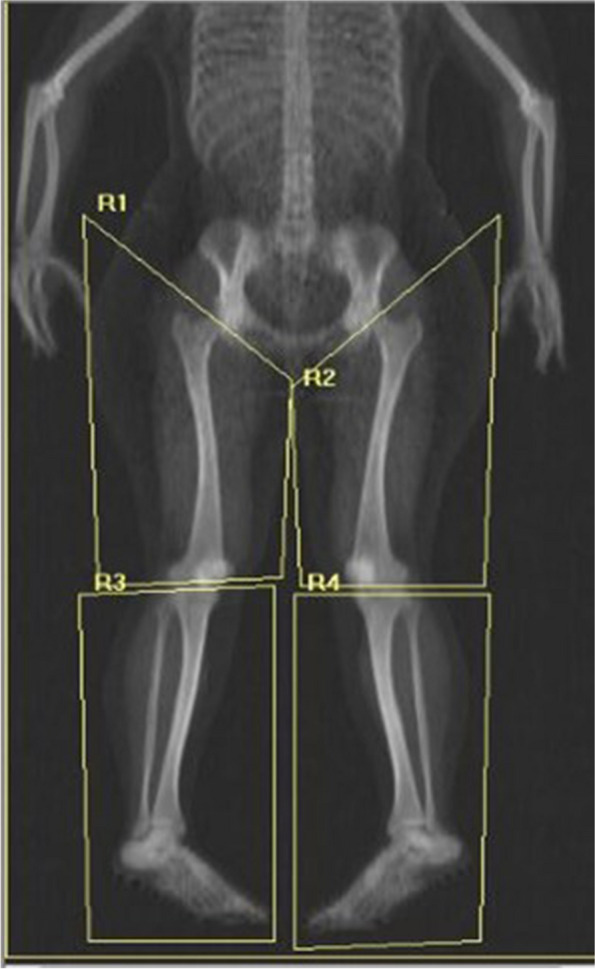
An example of body regions of the lower extremities manually drawn by an observer

The observers that analyzed the scans were medical interns at the time of analyzing the scans and had no previous experience with analyzing DXA scans. Both observers received a basic training under supervision of an experienced technician. Both legs were segmented into an upper and lower part, adding up to a total of four body regions. The upper border from the upper region being the femur neck, the lower border from the upper region being the most distal part of the femur. For the lower region, the upper border was the most proximal part of the tibia, and the lower border was the most distal part of the calcaneus. An example is displayed in Fig. 1. After manually drawing the regions, the body composition of the designated regions is calculated by the Hologic APEX software, version 5.6.0.5 (HOLOGIC, Inc, Bedford MA, United Kingdom), according to the NHANES reference database. Researchers were blinded for clinical data of the patient and for the previous results.

### Sample size

For the current analysis, a consecutive series of 34 women included in the *BENEFIT* study from November 2019 until March 2020 were analyzed. A post hoc power analysis was performed to calculate the minimal detectable correlation.

### Statistical analysis

Descriptive statistics were used to describe the study population and their body composition. Inter-observer variability was calculated from the absolute variability from the first round of assessments. The mean relative difference between observers in the muscle mass measured was shown in a Bland–Altman plot with 95% limits of agreement (± 1.96 SD). The intraclass correlation coefficient (ICC) was calculated to evaluate the consistency between the two observers [20, 21].

Intra-observer variability was expressed as the absolute and relative difference within the two observers. The mean difference with 95% limits of agreement (± 1.96 SD) was shown in a Bland–Altman plot and the ICC was calculated to evaluate consistency within the two observers.

Statistical tests were performed using IBM SPSS version 26.0 for Windows (Armonk, NY, USA). Significance was assumed when the two-sided *p*-value < 0.05. The ICC was regarded as good when > 0.75.

## Results

### Participant characteristics

A consecutive series of 34 DXA scans were available for analysis in this study. None of the consecutively included participants were excluded from this analysis. The characteristics of the study population are shown in Table 1. All participants were female *BRCA1/2* GPV carriers who underwent RRSO at premenopausal age. Participants included in this study had a median age of 58 years (interquartile range (IQR) 53–65). Median duration of menopause was 13.5 years (IQR 12.8–18.0). Median body mass index (BMI) was 24.6 kg/m^2^ (IQR 21.7–29.7). Table 2 shows the mean and median of the muscle mass of the study population of all four measurements and per observer. The mean muscle mass of the lower limbs of the study population was 14.1 ± 3.1 kg, measured at 13.8 ± 3.0 kg by observer 1 and 14.3 ± 3.1 kg by observer 2.

**Table 1:** Characteristics of the study population

*Study participants (n* = *34)*	Mean ± SD	Median (IQR)
Age (in years)	58.8 ± 7.2	58.0 (53.0–65.3)
Age at RRSO (in years)	43.7 ± 5.7	44.5 (38.0–49.0)
Duration of menopause (in years)	15.2 ± 3.6	13.5 (12.8–18.0)
Length (in cm)	167.7 ± 7.0	168.5 (163.0–173.0)
Weight (in kg)	73.6 ± 18.1	69.8 (60.0–80.0)
BMI (in kg/m^2^)	26.1 ± 5.6	24.6 (21.7–29.7)
Mean bone mass of the lower extremity (in g)	760 ± 87	763 (712–809)
Mean fat mass of the lower extremity (in kg)	12.1 ± 5.2 kg	10.7 (8.9–14.6) kg

*cm* centimeters, *g* grams, *IQR* interquartile range, *kg* kilograms, *RRSO* risk-reducing salpingo-oophorectomy, *SD* standard deviation

**Table 2:** Muscle mass of study population per observer

	**Mean ± SD**	**Median (IQR)**
Mean muscle mass of four measurements (in kg)	14.1 ± 3.1	13.2 (12.0–15.1)
Mean muscle mass as measured by observer 1 (in g)	13.8 ± 3.0	12.9 (11.8–14.9)
Mean muscle mass as measured by observer 2 (in g)	14.3 ± 3.1	13.6 (12.2–15.6)

*g* grams, *IQR* interquartile range, *kg* kilograms, *SD* standard deviation

### Inter-observer variability

**Fig. 2:**
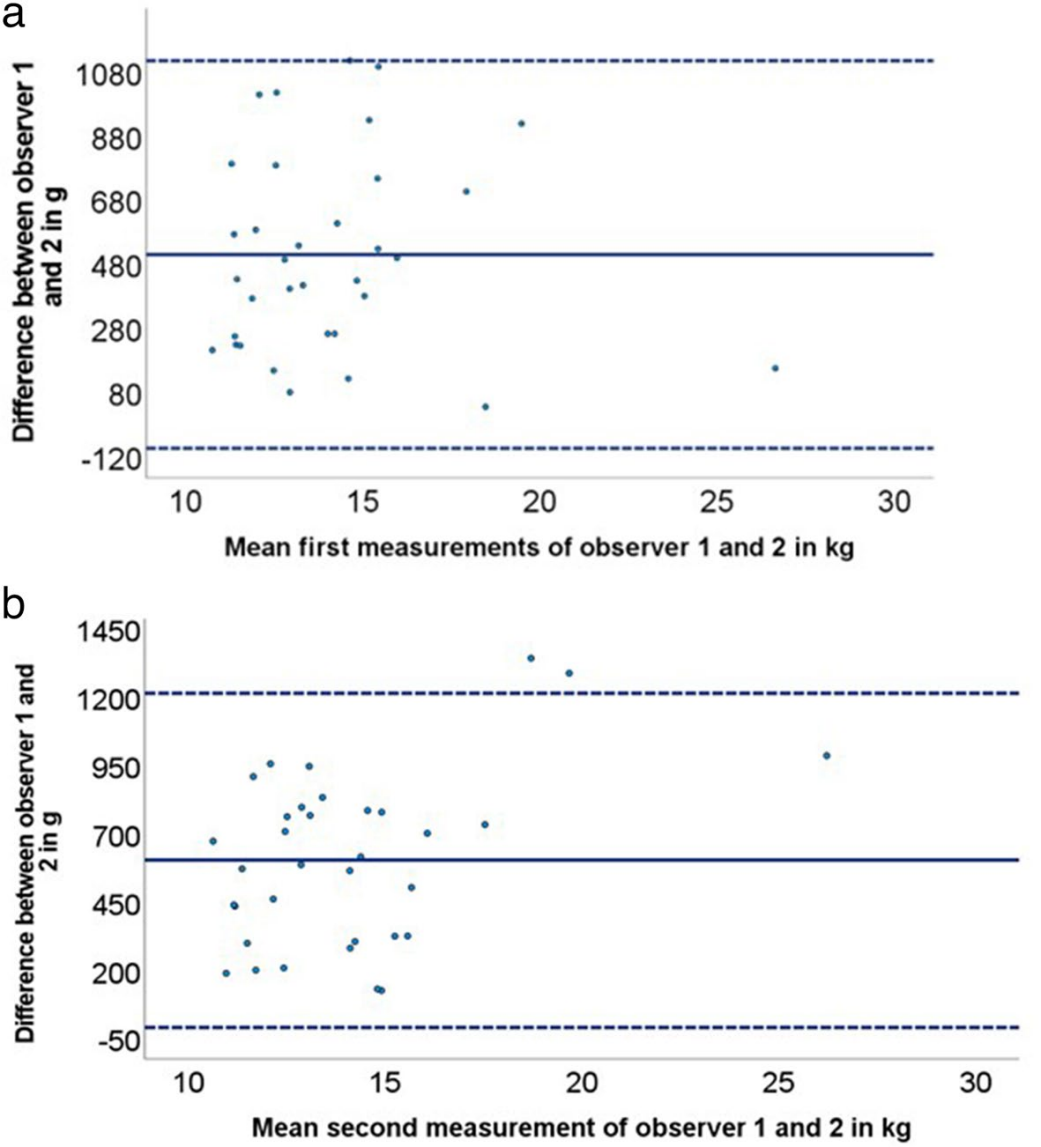
Bland–Altman plot of the interobserver variability, with (**a**) presenting the comparison between the first measurements of observer 1 and observer 2 and (**b**) presenting the comparison between the second measurements of observer 1 and observer 2. Bold blue lines present the mean difference and the 95% confidence interval of the mean, presenting the limits of agreement (dotted lines)

For the absolute inter-observer variability in the first round of measurements, the mean difference in muscle mass between the two observers was 530 ± 249 g (median 587, IQR 391–688). Relative inter-observer variability was 0.037 ± 0.022%. The ICC for the inter-observer variability was 0.997 (95% CI 0.994–0.998). Figure 2 shows the Bland–Altman plot for the inter-observer variability, for both the first and second round of measurements. It shows an equal distribution of values with no systematic difference or outliers.

### Intra-observer variability

**Fig. 3:**
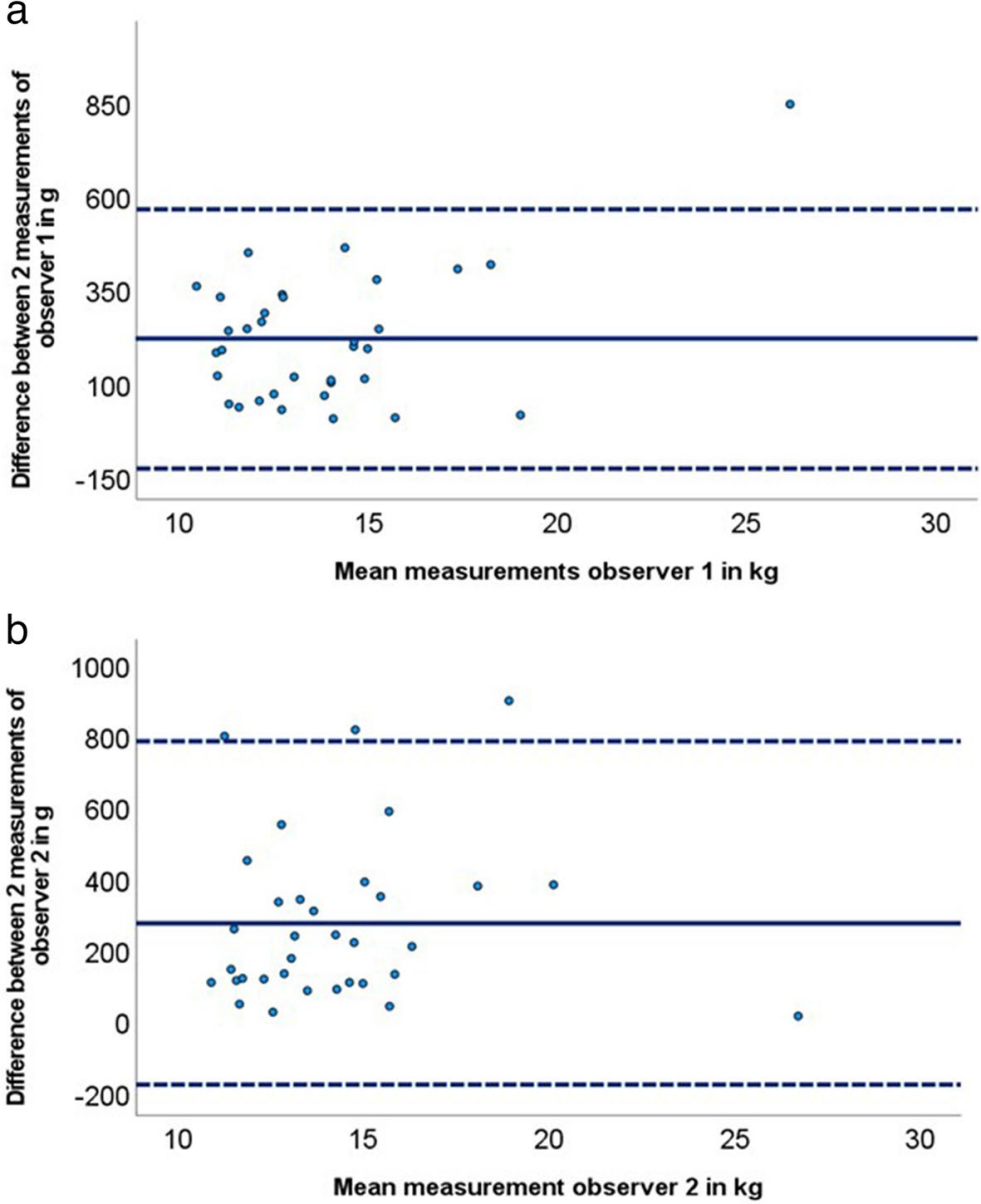
Bland–Altman plot of intra-observer variability, with (**a**) performed by observer 1 and (**b**) performed by observer 2. Bold blue lines present the mean difference and the 95% confidence interval of the mean, presenting the limits of agreement (dotted lines)

The mean difference between the analyses of observer 1 was 215 ± 177 g and for observer 2 270 ± 230 g. Relative inter-observer variability of observer 1 was 0.019 ± 0.016% and of observer 2 0.016 ± 0.011%. The ICC for the intra-observer variability of observer 1 was 0.998 (95% CI 0.996–0.999). The ICC for observer 2 was 0.997 (95% CI 0.993–0.998). Figure 3 shows the Bland–Altman plot for the intra-observer variability. For both observers, no significant systematic difference was seen. Both observers showed acceptable limits of agreements and a minimal number of outliers.

### Costs and radiation dose of DXA, CT, and MRI

The total cost of a single whole DXA scan amounted to €62, for CT €77, and for MRI €195 (see Table 3). The major cost driver for all three methods was personnel, presenting about 50% of the costs.

**Table 3:** Duration and costs of the different types of scans, subdivided for staff, equipment, and overhead and housing costs [18, 19]

	**DEXA**	**CT**	**MRI**
Scan duration (min)	15	10	45
Reading time (min)	5	5	5
Staff costs (€)	34	34	101
Equipment (€)	12	22	44
Overhead and housing (€)	16	20	51
Total costs (€)	62	77	195

The effective radiation dose of CT measuring the muscle mass of the lower extremities is estimated at 2.5–3.0 mSv [22], whereas this is approximately 4.2 µSv for a DXA WBC and 2 µSv for the lower extremities [23]. For MRI, no radiation is used.

### Power

Assuming a power of > 0.8, a significance level of 0.05, and our sample size of 34 women, the minimal detectable correlation was 0.48.

## Discussion

The aim of this study was to evaluate the inter- and intra-observer variability of DXA and to compare the costs and the radiation dose of DXA to CT and MRI in measuring muscle mass of the lower limb in surgically menopausal women. Inter-observer variability had a good ICC of 0.997 (95% CI 0.994–0.998). With regard to intra-observer variability, observer 1 had an ICC of 0.998 (95% CI 0.996–0.999) and observer 2 had an ICC of 0.997 (95% CI 0.993–0.998). Calculated based on the guidelines for economic evaluation of the Dutch National Health care institute, costs for DXA were €62, for CT €77, and for MRI €195. The estimated radiation dose of CT is 2.5–3.0 mSv, for DXA 2–4 µSv.

The reliability of calculating muscle mass with DXA in this study is comparable to the reliability of CT and MRI found by Sinelnikov et al. for measuring spinal muscle mass [24]. The ICC for inter-observer variability was 0.946 for CT and 0.957 for MRI, with an intra-observer variability of 0.988 and 0.968 for CT and 0.986 and 0.979 for MRI. In a study by Chen et al. in women with a mean age of 70.7 years, lean soft tissue mass derived from DXA highly correlated with MRI-derived skeletal muscle mass (*r* = 0.94, *p* < 0.001) [25]. In addition, DXA-measured thigh fat-free mass was highly correlated to thigh muscle volumes with multislice CT (*r* = 0.96, *p* < 0.0001) [26].

Another method to assess muscle mass and/or diagnose sarcopenia is BIA. Cheng et al. found BIA to overestimate skeletal muscle mass in comparison to DXA in older adults [27], whereas Peppa et al. showed strong agreement between BIA and DXA with minimal proportional differences of no clinical significance in overweight/obese postmenopausal women [28]. Compared to 24-h urinary creatinine excretion, which is considered a gold standard to measure total skeletal muscle mass, DXA may overestimate muscle mass because it does not differentiate between water and bone-free lean mass [29]. Nonetheless, correlation between muscle mass estimation by DXA and urine creatinine is still high (*r* = 0.80) [30]. In addition, collection of 24-h urine to determine creatinine excretion is highly dependent on subject compliance in the collection process, which increases variability [30].

An advantage of DXA as opposed to CT and MRI is the lower costs. The relatively high costs for MRI are largely attributable to the higher personnel costs resulting from the longer scan time (45 min for MRI vs. 15 min for DXA and 10 min for CT), but overhead and housing costs are also higher for MRI. Although CT has a shorter scan time, it is €15 more expensive and, more importantly, is accompanied with a higher radiation dose: 2.5–3.0 mSv vs. 4.2 µSv. In addition, DXA is the gold standard to diagnose osteoporosis [31, 32]; therefore, one type of scan could be used to diagnose radiologic sarcopenia and osteoporosis in an efficient manner.

To the best of our knowledge, there is no published study that compares reliability, costs, and radiation exposure of DXA to CT and MRI in the measurement of muscle mass. Assuming a power of > 0.8, a significance level of 0.05, and our sample size of 34 women, the minimal detectable correlation was 0.48, making the sample size sufficient to determine the reliability of DXA. A strength of this study is the standardized protocol used in the analysis of DXA, thereby minimizing measurement bias. Observers were blind to clinical data, which minimized attribution bias and framing bias.

However, recall bias may have influenced our results, as time in-between the two assessments was 2 to 3 h. It was not possible to prolong time in-between the two assessments due to COVID-19 regulations. We estimate that this had a minor effect on our outcomes, because analyzing the scan requires manual drawing at each attempt, a procedure that is difficult to replicate exactly. Additionally, no direct comparison could be made between the ICCs found in this study to those of CT and MRI, because only literature on spinal muscle mass was available [24]. In future research, a direct comparison of the reliability of DXA, CT, and MRI should be made in a study population with a variety in age and gender. Subsequently, diagnostic cut-off values can be determined for diagnosing radiologic sarcopenia with DXA.

## Conclusions

To conclude, the inter-observer variability of DXA in the analysis of muscle mass of the lower extremities in middle-aged women is low. The intra-observer variability was somewhat higher than the inter-observer variability, but still excellent. Additionally, costs and radiation dose are low for DXA, and therefore, DXA is a suitable alternative to CT and MRI in the diagnosis of radiologic sarcopenia. More research is needed to compare the reliability of DXA directly to CT and MRI in different populations and to determine age- and gender-specific diagnostic cut-off values.

### Supplementary Information

**Supplementary Material 1.**

## Data Availability

The datasets used and/or analyzed during the current study are available from the corresponding author on reasonable request.

## Abbreviations

BIA: Bioelectrical impedance analysis
BMD: Bone mineral density
BMI: Body mass index
GPV: Germline pathogenic variant
ICC: Intraclass correlation coefficient
RRSO: Risk reducing salpingo-oophorectomy
WBC: Whole-body composition
