# Supplementary material for: Reliability, costs, and radiation dose of dual-energy X-ray absorptiometry in diagnosis of radiologic sarcopenia in surgically menopausal women
Source: Insights Imaging. 2024 Apr 8;15:104. doi: 10.1186/s13244-024-01677-w (PMC11001834; doi:10.1186/s13244-024-01677-w)
Supplement: Supplementary file 1 — Supplementary Material 1. [file 13244_2024_1677_MOESM1_ESM.pdf]

Projectverantwoordelijke: klinisch
Groep patiënten: Bepalen van de BMD en de BCA (Body Composition Analyses = van de weke delen de vetmassa en de niet-vet massa bepalen).

---------- **VOORBEREIDING PATIËNT** --

Geen

---------- **VOORBEREIDING ONDERZOEK** --

Patiënt: De patiënt alle kleding uit laten trekken op onderbroek en sokken na (eventueel mag hemd aanblijven). Alle sieraden, piercings en haarspeldjes moet uitgedaan worden. Bij een BCA meting worden ook de weke delen gemeten. Hierbij is het van belang dat zoveel mogelijk kleding is verwijderd, dit draagt anders bij aan de weke delen meting.

DEXA: Verwijder op de tafel alle attributen ook het hoofdkussen. Voor de hygiëne mag een laken op tafel liggen.

---------- **RADIOFARMACON** --

Geen

---------- **ACQUISITIE** --

Camera: DEXA

Protocol: Whole Body

Positionering volwassene: Zorg dat de patiënt recht en in het midden van de tafel ligt. Handen naast het lichaam met de vingers licht gespreid. Voeten enigszins in endorotatie . Controleer boven en ondergrens van het scangebied. Het gehele lichaam moet gescand worden.
Indien patiënt te lang is, de patiënt zo hoog mogelijk op tafel leggen, zodat het hoofd in z'n geheel wordt gescand. Iets van de voeten missen geeft maar een kleine fout in de meting (de knieën **NIET** laten optrekken)
Wanneer patiënt te breed is plaats de handen verticaal. Indien dit niet voldoende is, de patiënt iets uit het midden leggen zodat de **rechterarm** goed in het scanveld ligt en linkerarm er net buiten het scanveld. Bij het uitwerken komt de vraag of de rechterarm naar de linkerarm gekopieerd moet worden. Dit toestaan.
Laat de patiënt naar het plafond kijken.

Positionering kind: Laat de patiënt omhoog schuiven naar het hoofdeinde en meet daarna met de rolmaat de benodigde scanlengte. Voer dit in voordat je de scan start (anders kan de scanlengte een volgende keer niet verkleind worden, vergroten kan wel).

Start de scan: Controleer of de patiënt goed stil blijft liggen.
De scan wordt van craniaal naar caudaal in drie gedeelten gescand.
Controleer of er zo identiek mogelijk gescand wordt ten opzichte van de baseline scan.

---------- **PROCESSING** --

Er wordt een analyse van apart armen, benen, hoofd en rug gedaan.
Controleer de auto-analyse.

© Nadruk verboden

Regions:
Aan de hand van een vijftal regio's wordt de analyse berekend
1. hoofd
2. arm van romp gescheiden
3. ruggenwervels
4. bekkenrand
5. omgekeerde driehoek

Bepaal de vijf regio's op de volgende manier:
Line Mode
1. Selecteer de volgende **horizontale lijnen** en plaats deze als volgt (zie afbeelding 1):
    a. ondergrens hoofd
    b. grens TWK-LWK
    c. bovenrand bekkenkam

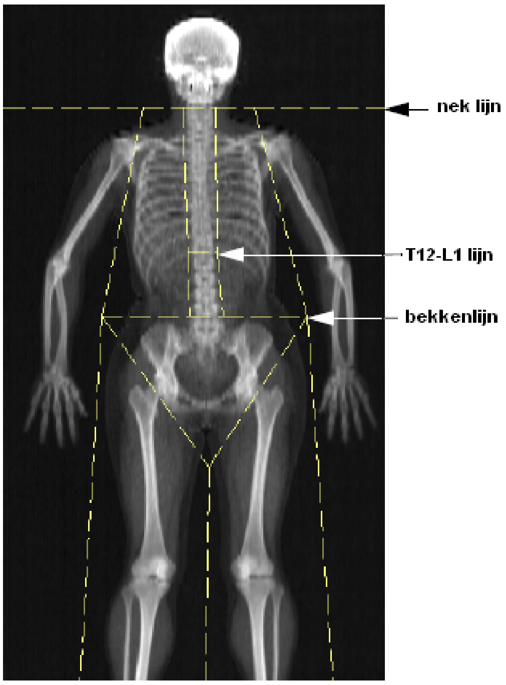

Afbeelding 1

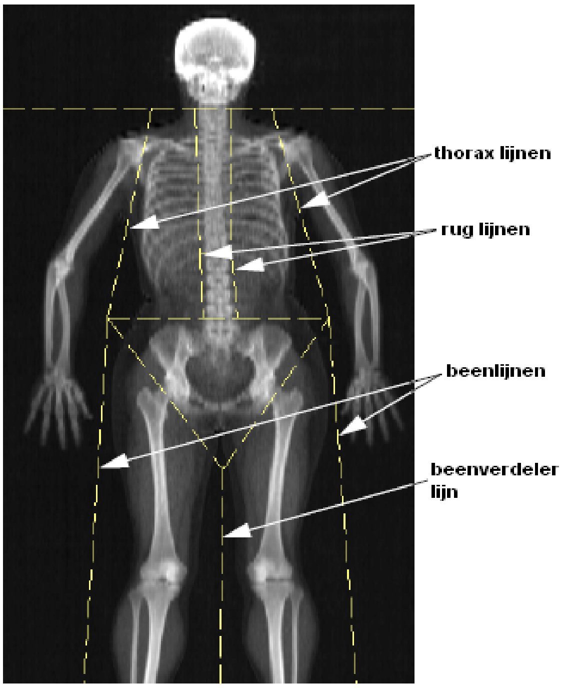

Afbeelding 2

2. Selecteer de volgende **verticale lijnen** en plaats deze als volgt (zie afbeelding 2):
    a. tegen de borst aan
    b. tegen de wervelkolom
    c. tegen de benen (inclusief weke delen)
    d. evenwichtige scheidingslijn tussen de benen

Via **Point Mode** worden de verticale lijnen verfijnder geplaatst (zie afbeelding 3).
3. Fine tune de **verticale lijnen**
    a. Selecteer het punt bij de linker schouder en plaats deze in het glenoid, zodat humerus en scapula gescheiden zijn. Evenzo rechter schouder.
    b. Selecteer de linker punten naast de wervelkolom en plaats dit gelijk aan de kromming van de wervelkolom. Evenzo voor rechter zijde.

© Nadruk verboden

   c. Selecteer en verplaats de punten zodanig dat ook de weke delen inclusief zijn
   d. Punt van de omgekeerde driehoek verplaatsen zodat de scheidingslijn door het midden van de femurhals gaat.
   e. Deze punten zodanig plaatsen dat zoveel mogelijk weke delen bij de heupen wordt ingesloten, maar zonder de vingers.

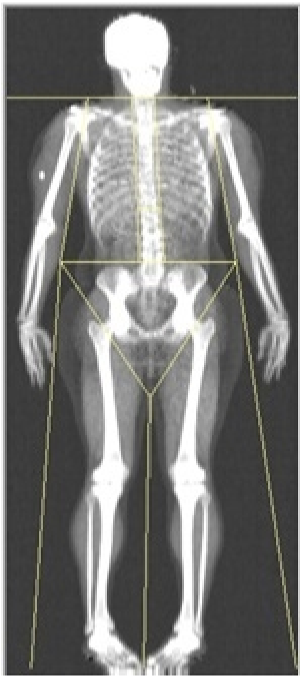

Afbeelding 3

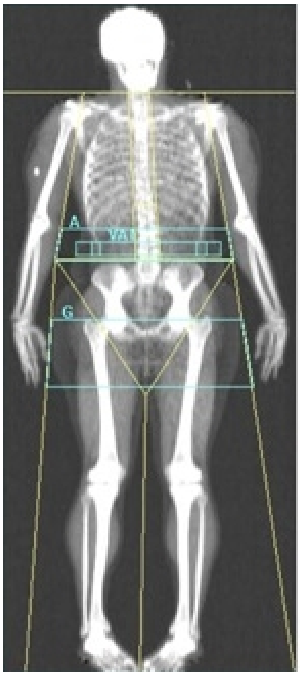

Afbeelding 4

4. **A/G regions**
   Deze regio's worden automatisch juist geplaatst op basis van de horizontale lijnen bovenrand bekkenkam, ondergrens hoofd en de verticale thoraxlijnen (zie afbeelding 4).
   a. **Android regio:**
      a. Ondergrens: snijlijn bekkenkam
      b. Hoogte: 20% van de afstand snijlijn bekkenkam en ondergrens hoofd.
      c. Breedte: snijlijnen armen
   b. **Genoid regio:**
      a. Bovengrens: 1.5x hoogte android regio, gerekend vanaf de bekkenkam snijlijn
      b. Hoogte: 2x hoogte android regio
      c. Breedte: snijlijnen armen

© Nadruk verboden

c. De VAT regio, Visceral Adipose Tissue, wordt automatisch geplaatst.
De VAT regio is het vet in de buikholte, binnen de abdominale spierwand. Het vet tussen de buikorganen.

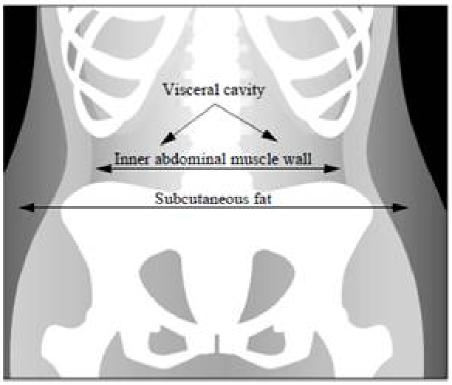

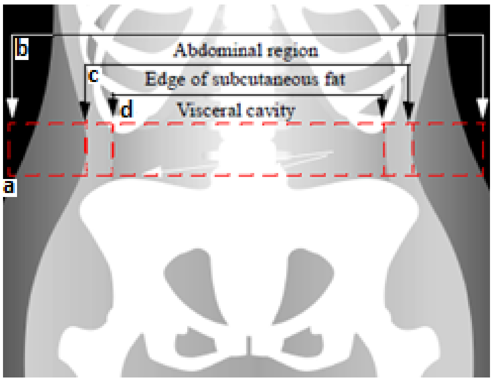

a. De VAT regio mag geen bot van het bekken bevatten
b. De buitenste set laterale lijnen zijn geplaatst op de huidlijn.
c. De volgende set laterale lijnen in de abdominale regio zijn geplaatst aan de buitenkant van het subcutane vet.
d. De daarop volgende set laterale lijnen zijn geplaatst aan de binnenzijde van het subcutane vet. Deze lijnen vormen de begrenzing voor de viscerale holte.

Results[1]
BMD resultaten met bijbehorende T en Z-scores worden weergegeven.
BCA resultaten worden weergegeven met bijbehorende T en Z-scores.

---------- **BIJZONDERHEDEN** ---

- Bij metaal in het bekken/been waarden <u>niet</u> kopiëren naar het andere been.

[1] Analyseren en Rate of Change Rapport (ROC)
Vrijdag 20 februari 2012 is er nieuwe software in de Discovery gekomen. Nu kan het ROC rapport voor een whole body niet geprint worden, omdat de oude scans met de oude software zijn geanalyseerd. Om de uitslag samen met de vorige scan te laten zien, moet deze oude scan opnieuw geanalyseerd worden.
De afspraak is: de laatste whole body die gemaakt is (voor 10-02-2012) opnieuw te analyseren.
Deze whole body's moeten opnieuw gearchiveerd worden.

Aan de hand van de NHANES referentie database worden BMD waarden, niet-vet massa (oppervlakte, regionaal en totaal) bepaald. Tevens wordt het percentage vet (regionaal, totaal, android en gynoid) weergegeven.
Binnen de android regio wordt het viscerale vetweefsel (VAT) gemeten. Als resultaat wordt de viscerale vetregio en massa met het bijbehorende volume getoond.

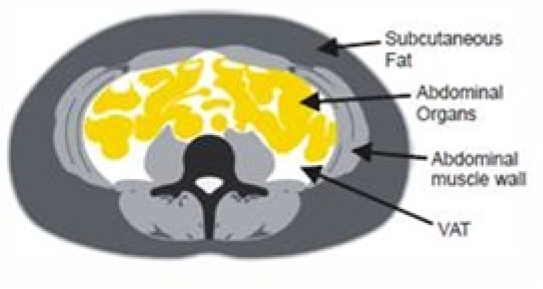

© Nadruk verboden
